# Robust Volume Assessment of Brain Tissues for 3-Dimensional Fourier Transformation MRI via a Novel Multispectral Technique

**DOI:** 10.1371/journal.pone.0115527

**Published:** 2015-02-24

**Authors:** Jyh-Wen Chai, Clayton C. Chen, Yi-Ying Wu, Hung-Chieh Chen, Yi-Hsin Tsai, Hsian-Min Chen, Tsuo-Hung Lan, Yen-Chieh Ouyang, San-Kan Lee

**Affiliations:** 1 Department of Radiology, Taichung Veterans General Hospital, Taichung, Taiwan; 2 College of Medicine, China Medical University, Taichung, Taiwan; 3 Department of Radiological Technology, Central Taiwan University of Science and Technology, Taichung, Taiwan; 4 Department of Biomedical Engineering, Hung Kuang University, Taichung, Taiwan; 5 Center of Quantitative Imaging in Medicine, Department of Medical Research, Taichung Veterans General Hospital, Taichung, Taiwan; 6 Department of Psychiatry, Taichung Veterans General Hospital, Taichung, Taiwan; 7 Department of Electrical Engineering, National Chung Hsing University, Taichung, Taiwan; National Yang-Ming University, TAIWAN

## Abstract

A new TRIO algorithm method integrating three different algorithms is proposed to perform brain MRI segmentation in the native coordinate space, with no need of transformation to a standard coordinate space or the probability maps for segmentation. The method is a simple voxel-based algorithm, derived from multispectral remote sensing techniques, and only requires minimal operator input to depict GM, WM, and CSF tissue clusters to complete classification of a 3D high-resolution multislice-multispectral MRI data. Results showed very high accuracy and reproducibility in classification of GM, WM, and CSF in multislice-multispectral synthetic MRI data. The similarity indexes, expressing overlap between classification results and the ground truth, were 0.951, 0.962, and 0.956 for GM, WM, and CSF classifications in the image data with 3% noise level and 0% non-uniformity intensity. The method particularly allows for classification of CSF with 0.994, 0.961 and 0.996 of accuracy, sensitivity and specificity in images data with 3% noise level and 0% non-uniformity intensity, which had seldom performed well in previous studies. As for clinical MRI data, the quantitative data of brain tissue volumes aligned closely with the brain morphometrics in three different study groups of young adults, elderly volunteers, and dementia patients. The results also showed very low rates of the intra- and extra-operator variability in measurements of the absolute volumes and volume fractions of cerebral GM, WM, and CSF in three different study groups. The mean coefficients of variation of GM, WM, and CSF volume measurements were in the range of 0.03% to 0.30% of intra-operator measurements and 0.06% to 0.45% of inter-operator measurements. In conclusion, the TRIO algorithm exhibits a remarkable ability in robust classification of multislice-multispectral brain MR images, which would be potentially applicable for clinical brain volumetric analysis and explicitly promising in cross-sectional and longitudinal studies of different subject groups.

## Introduction

Quantitative volume assessment of brain tissues from MR images is an important and accessible means to yielding unique insight into understanding and diagnosis of normal and diseased brains. Multispectral MRI provides a stack of high quality images with excellent contrast and unique features of normal and pathological tissues, which are explicitly practicable in clinical diagnosis of diseases as well as the analysis of brain segmentation for volume quantification [1–4]. According to the particular algorithms used for brain tissue segmentation, the techniques can be categorized into several groups, region-based, contour-based and classification-based approaches [5]. Region- and contour-based approaches perform segmentation by partitioning a grey-scale image into a finite number of different tissue regions. However, in real cases, there exist significant overlaps between different tissue types due to biological tissue anomalies and imaging systematic factors, which could obviously hinder the practicability in clinical applications [6].

Currently, some high-level algorithms have been proposed to automatically perform more feasible segmentation of multispectral brain MRI by incorporating readily available *a priori* knowledge to support the unsupervised task. One of such high-level algorithm is an atlas-based approach which is efficient and capable of yielding accurate and consistent segmentation of multispectral brain MRI [7–9]. This approach basically constructs a standard atlas for the proper labeling of anatomical structures from *a priori* anatomical information and transforms the regions labeled in the atlas to the space of target images [10]. The use of the probabilistic atlas in brain segmentation involves two major processes of atlas construction, proper labeling the anatomical structures from the template images and image registration by transforming the atlas objects to the space of target images. As it is known, there exist potential sources of error in accurate segmentation of brain MRI [11, 12], and this technique may not be desirable for various challenging populations [9]. Another widely used segmentation tool, FAST of FSL (FMRIB software library) is based on a hidden Markov Random Field (MRF) model and an associated expectation-maximization algorithm [13]. MRF model uses spatial information to aid in classification by characterizing coherence in local neighborhoods [14,15]. The technique can provide a reproducible segmentation, particularly for image data that are noisy and ambiguous [16]. However, owing to the high computational complexity of these high-level segmentation techniques, this approach needs a long processing time [17].

In order to achieve optimal brain segmentation, a new supervised approach derived from multispectral remote sensing techniques has been developed for multislice-multispectral brain MRI. The proposed technique implements an iterative version of Fisher’s linear discriminant analysis (FLDA) coupled with the independent component analysis (ICA) and support vector machine (SVM) where SVM and FLDA are widely discriminative classifiers in pattern classification but developed from completely different design rationales in terms of how to use training samples. The TRIO of ICA, SVM and FLDA combines the strengths of these three individual techniques to produce best possible classification of brain tissues via only a small manually selected set of training samples from multislice-multispectral MRI data. Additionally, the proposed method provides unique benefit of operating classification in the native coordinate space, which not only avoids the registration problems in transformation to a standard coordinate space, but also preserves the high spatial-resolution image profiles without smoothing filtering during the coordinate transformation processing. In the previous report, it has been shown that the method was a promising technique in classification of 2DFT multislice-multispectral MRI of normal synthetic and real normal brain data [18]. For the experiments conducted in this paper, we extended to produce brain volume measurements of gray matter (GM), white matter (WM) and cerebral spinal fluid (CSF) in brain volume morphometry of 3-dimensional Fourier transformation (3DFT) high spatial resolution multislice-multispectral MRI. The accuracy and reproducibility were tested by performing experiments using the synthetic normal brain data. For clinical MRI data with no available gold standard for comparison in the in vivo experiments, the variability was analyzed to evaluate its reproducibility of quantification results in normal young adult, normal aged adult, and dementia, and illustrate the effectiveness of the proposed method in different study groups.

## Materials and Methods

### Synthetic Images

The synthetic MRI data from the BrainWeb Database at the McConnell Brain Imaging Centre of the Montreal Neurological Institute (MNI), McGill University (http://www.bic.mni.mcgill.ca/brainweb) was utilized to objectively test the accuracy and reproducibility of the TRIO algorithm classifier. The synthetic normal images include 90 axial slices of the pre-computed simulated brain database, T1-weighted imaging (T1WI), T2-weighted imaging (T2WI), and proton density imaging (PDI) data with 1mm isotropic voxel size. Seven data sets of the synthetic image data were chosen with 4 noise levels of 0%, 1%, 3%, and 5% (calculated relative to the brightest tissue) and two intensity non-uniformity levels of 0% and 20%.

### Clinical Brain MRI

Clinical brain MRI data were acquired from a whole body 1.5 T MRI system (Aera, Siemens, Erlangen, Germany) with a phase-array head coil. Three study groups consisted of ten young healthy subjects (5 male, 5 female; 21.6±0.9 years old), ten healthy elderly subjects (7 male, 3 female; 58.7±9.0 years old) and ten dementia patients (3 male, 7 female; 71.2±9.3 years old). The inclusion criteria of dementia were based on the Diagnostic and Statistical Manual of Mental Disorders—IV. The exclusion criteria were presence of white matter lesions, larger than grade 2 of the visual Fazekas scale [19]. The Institutional Review Board of Taichung Veterans General Hospital, Taichung reviewed and approved the experimental protocol and the consent procedure. Written informed consent was obtained from all volunteers and patients.

The imaging protocol included three high-resolution 3DFT acquisition sequences: T1WI with magnetization-preparation rapid acquisition gradient echo (MP-RAGE; repetition time, TR = 2800ms; echo time, TE = 3.98mm; inversion time, TI = 930ms; flip angle = 6°), T2WI (TR = 3000ms; TE = 280ms; echo train length, ETL = 190) and fast fluid-attenuated inversion-recovery (FLAIR; TR = 5000 ms; TE = 350 ms; TI = 1800 ms; ETL = 242) with SPACE (Sampling Perfection with Application Optimized Contrasts using different Flip Angle Evolutions) technique. The recently evolved 3-D turbo spin echo sequence could provide a high spatial resolution image with using variable flip angle evolutions, which allows for longer echo trains and optimal T2 contrast with longer TE. Other imaging parameters were voxel size 1x1x1mm, matrix = 256x256x176, number of excitation (NEX) = 1.

### Description of Proposed Algorithms

In dealing with multispectral data of a remote sensing image, FLDA, a multiple-class classifier can effectively solve the problems with a mathematically simple and robust method [20, 21]. However, FLDA, notably being a powerful supervised classifier, needs a sufficiently large pool of training samples to reflect the global properties of the class distributions in order to produce reliable classification. Such a method may generally suffer from large measurement variability and be also difficult in access to accurate class labels of a large number of training data [20]. To resolve this issue, the SVM classifier is considered as a preprocessing technique of FLDA for providing a larger pool of training data with enough brain tissue properties to initiate an iterative version of FLAD for a consistent classification. SVM only requires a small set of training samples as support vectors which can effectively minimize the operating task and allow its clinical practicability. However, SVM could only achieve high performance in classification of brain MRI under the conditions of appropriate selection of nonlinear kernels and optimal parameters. The ICA in our proposed TRIO algorithm method has been particularly developed to enhance the image contrast and can be used as a preprocessing technique to separate different brain tissue structures. Specifically, ICA not only significantly improves the accurate classification of SVM without using optimal parameters or specified kernels.

**Independent Component Analysis**. The ICA approach can be considered as a preprocessing method to enhance the image contrasts of GM, WM, and CSF by removing the 1^st^ and 2^nd^ statistics of the MR image data for further separating different brain tissue structures in a set of statistically independent components [22, 23]. For the experiments conducted here, the entire image data with M multiple slices were stacked as a cube, **I** = ${\left\{{\text{I}}_{i}\right\}}_{i=1}^{\text{M}}$, with each individual single slice I_*i*_ = (T1, T2, FLAIR). Assume that **x** is a pixel vector in a single MR image slice *I* which is linearly mixed by a set of *p* statistically independent signal sources, *s*_1_, *s*_2_, …, *s*_*p*_ by means of a mixing matrix **A** as:

x=As (1)

where A is an *L*×*p* mixing matrix and s is a *p*-dimensional signal source vector s = (s_1_, s_2_, …, s_*p*_)^*T*^ of brain tissue clusters needed to be separated. The goal of the ICA is to unmix the observed mixed signal source **x** via equation (7) by finding an unmixing matrix **W** by which the *p* unknown signal sources representing brain tissues present in the signal source vector **s** can be separated through the following unmixing equation:

s=Wx (2)

**Support Vector Machine**. SVM, a classification-based discriminant analysis, makes use of a nonlinear kernel to map the original data space into a higher dimensional feature space to address the issue of linear inseparability where SVM attempts to find an optimal hyperplane that separates two classes of data samples as far as possible by maximizing the margin of separation between classes and the hyperplanes [24, 25]. Based on the learning principle of structural risk minimization, SVM is capable to perform well with the worse training examples, called support vectors, which are difficult to classify. Its major strengths are that the approach significantly reduces the computational complexity and requires relatively small support vectors with no need of training data statistics [26]. This advantage is very useful in reducing the large scale learning task and minimizing human intervention and the operating burden in manually labeling the target tissues. Nevertheless, SVM could only achieve high performance in classification of brain MRI under appropriate cost and gamma parameters of a RBF kernel. After ICA preprocessing to enhance the image contrast, the effect of optimal parameters could be definitely mitigated [27]. Our previous results also illustrated that SVM could perform well for one single slice of multispectral MRI data at a time, but not multislice-multispectral MRI data.

**Fisher’s Linear Discriminant Analysis**. FLDA is widely used in statistical pattern recognition and machine learning to find a set of features to characterize patterns to be analyzed [20, 21, 28]. FLDA’s strength in pattern classification lies on the criterion used for optimality, which is called Fisher’s ratio defined by the ratio of between-class scatter matrix to within-class scatter matrix. More specifically, assume that there are *n* training sample vectors, ${\left\{r_{i}\right\}}_{i=1}^{n}$for *p*-class classification, *C*_1_, *C*_2_, ⋯, *C*_p_ with *n*_*j*_ being the number of training sample vectors in the *j*-th class *C*_*j*_. Let μ be the global mean of the entire training sample vectors, denoted by $\mu =\frac{1}{n}\sum_{i=1}^{n}r_{i}$ and μ _*j*_ be the mean of the training sample vectors in the *C*_*j*_, denoted by$\mu_{j}=\frac{1}{n_{j}}\sum_{r_{i}\in C_{j}}r_{i}$. The within-class scatter matrix, **S**_*W*_, between-class scatter matrix **S**_*B*_, and total scatter matrix are defined by Duda in 2001 [20] as follows.

$$S_{W}=\sum_{j=1}^{p}S_{j}\text{ where }S_{j}=\sum_{r\in C_{j}}\left(r-\mu_{j}\right){\left(r-\mu_{j}\right)}^{T}$$ (3)

$$S_{B}=\sum_{j=1}^{p}n_{j}\left(\mu_{j}-\mu \right){\left(\mu_{j}-\mu \right)}^{T}$$ (4)

$$S_{T}=\sum_{i=1}^{n}\left(r_{i}-\mu \right){\left(r_{i}-\mu \right)}^{T}=S_{W}+S_{B}.$$ (5)

By virtue of (3) and (4), Fisher’s ratio is then defined by

$$\frac{x^{T}S_{B}x}{x^{T}S_{W}x}\text{over vector}x.$$ (6)

The goal of the FLDA is to find a set of feature vectors that maximize Fisher’s ratio specified by (6). The number of feature vectors found by Fisher’s ratio is determined by the number of classes to be classified, which is *p*-1 because the rank of **S**_*B*_ in (6) is *p*-1. More details can be referred to Bishop [29] and Chang [30].

**Fig 1:**
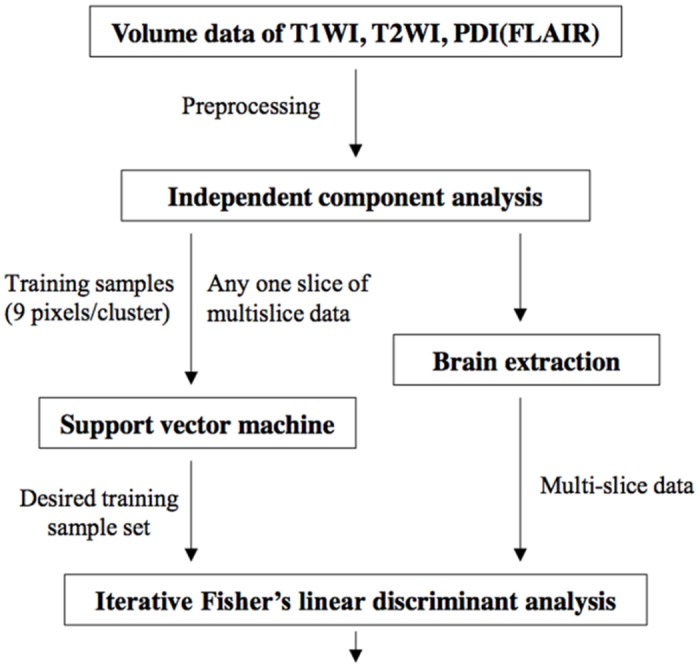
Flow chart of the hybrid classifier, coupling ICA, SVM and IFLDA for brain MRI classification and segmentation. First, the pre-processing step included registering FLAIR and T2WI with T1WI and correcting intensity inhomogeneity correction using N3 method. Second, the entire volume data of multislice-multispectral MR image data are automatically sphered to be a new data set by using ICA to remove the first two order statistics. Third, a small set of training data, containing a 3x3 matrix (of 9 pixels) of GM, WM, CSF, and background (BG) was manually identified by operators from a specific image slice of 3D images for SVM classification of the sphered multispectral images. At the same time, all the sphered multispectral images go through skull striping with BET. Finally, the output of SVM serves as a large pool of training samples for initiation of an iterative version of FLDA,

**Image Data Processing**. The whole procedures of the TRIO algorithm method proposed in this paper consist of five stage processes (Fig. 1). First, the pre-processing step included motion correction with rigid body approach to registering FLAIR and T2WI with T1WI, intensity inhomogeneity correction using Non-parametric Non-uniformity intensity Normalization (N3) method and skull striping with FSL-Brain Extraction Tool (BET) [31]. The default BET parameters were used with fractional intensity threshold = 0.5 and threshold gradient = 0.0. Second, the entire volume data of multislice-multispectral MR image data, $I=\left[{\text{I}}_{\text{1}}{\text{, I}}_{\text{2}}\text{, }\cdots {\text{, I}}_{\text{M}}\right]$, are sphered by removing the first two order statistics to be a new data set of$J=\left[{\text{J}}_{\text{1}}{\text{, J}}_{\text{2}}\text{, }\cdots {\text{, J}}_{\text{M}}\right]$. Third, a small set of training data of GM, WM, CSF, and background (BG) (denoted$S_{i}^{\text{initial}}$), was manually identified by operators from a specific image slice I_*i*_ of 3D images for SVM classification of the sphered multispectral images. In the experiment, the training data size of 3x3 pixels was used for effective SVM classification according to the previous study of no significantly statistical difference between the 3x3 pixels of training samples and the larger sample sizes [27]. Let $S_{i}^{\text{SVM}}$ denote the set of the SVM-classified data samples in the specific image slice J_*i*_. Fourth, FLDA was implemented on the entire data set **J** of the multislice-multispectral images, using $S_{i}^{\text{SVM}}$ as a large pool of training samples from the specific image slice, J_*i*_, for linear discriminant analysis. Let $S_{I}^{\text{FLDA}}$ be the set of the FLDA-classified data samples. Finally, the classified results, $S_{I}^{\text{FLDA}}$, from FLDA were used as the training samples of the next FLDA to classify tissue substances iteratively.

**Performance Evaluation**. Similarity index was used to measure the “ground truth” classification of each GM, WM, and CSF voxel. Assume that X and Y are two data sets and the similarity index is defined

$$\text{SI}=\frac{2n\left(X\cap^{\text{​}}Y\right)}{n\left(X\right)+n\left(Y\right)}$$ (7)

where *n*(*X*) and *n*(*Y*) are the numbers of elements in set *X* and *Y*, ‘‘∩” is set union [32]. For synthetic data experiments, the accuracy of the TRIO algorithm in brain tissue classification was evaluated by comparing the classified GM, WM, and CSF voxels with the ground truth data using the similarity index. Brain volume measurements of GM, WM, and CSF from the real image data were repeated three times by one operator at an interval of one week. Brain volume measurements of GM, WM, and CSF from the real image data were repeated three times by a senior radiologist at an interval of one week for testing the intra operator variability. The inter operator variability was tested with three measurements by three operators, a senior radiologist, a senior researcher, and a medical student.

The experiments of the synthetic multispectral image segmentation were also performed by using the SPM8 (Statistical Parametric Mapping; Wellcome Department of Cognitive Neurology, Institute of Neurology, London) for comparison. Since there is no gold standard in the real MRI data, the intra- and inter-operator variability of brain volume measurements was obtained to evaluate the performance of the hybrid classifier.

## Results and Discussion

### Synthetic Image Data Analysis

The proposed TRIO algorithm effectively classified GM, WM, and CSF in synthetic MRI data with very high accuracy, sensitivity, specificity and similarity index, as shown in Table 1. The accuracies were in range of 0.992 to 0.997, the sensitivities in 0.969 to 0.996, the specificities in 0.996 to 0.998, and similarity indexes in 0.965 to 0.991 for the image data with 0% noise level and non-uniformity intensity. The performance was slightly decreased as the noise levels and the intensity non-uniformities were increased. The similarity indexes of GM and WM classification by using the TRIO algorithm were largely higher than those by using the SPM8 software, except for WM in the synthetic data with higher noise levels (Table 2). The results also demonstrated CSF classification, as good as those of GM and WM. The similarity indexes of the proposed method in CSF classification were much higher than those of the SPM8, illustrated in Table 2. In addition, the experimental results also revealed extremely low intra- and inter-operator variability in classification of GM, WM, and CSF in the synthetic multislice-multispectral MRI data.

**Table 1:** The results of GM, WM and CSF quantification in the high-resolution synthetic MRI (1x1x1mm^3^) at various parameter settings by using the trio-algorithm hybrid classifier.

	**Accuracy**			**Sensitivity**			**Specificity**			**Similarity index**		
	**GM**	**WM**	**CSF**	**GM**	**WM**	**CSF**	**GM**	**WM**	**CSF**	**GM**	**WM**	**CSF**
**n0rf0** ^**a**^	0.992	0.997	0.995	0.969	0.996	0.983	0.998	0.997	0.996	0.980	0.991	0.965
**n1rf0**	0.989	0.994	0.995	0.960	0.987	0.977	0.996	0.995	0.996	0.971	0.982	0.962
**n3rf0**	0.981	0.987	0.994	0.951	0.959	0.961	0.988	0.993	0.996	0.951	0.962	0.956
**n5rf0**	0.974	0.980	0.993	0.940	0.931	0.951	0.981	0.991	0.996	0.931	0.943	0.949
**n1rf20**	0.986	0.992	0.994	0.957	0.980	0.962	0.993	0.994	0.997	0.964	0.976	0.958
**n3rf20**	0.981	0.987	0.994	0.951	0.959	0.957	0.988	0.993	0.997	0.950	0.962	0.955
**n5rf20**	0.974	0.981	0.993	0.941	0.932	0.946	0.981	0.991	0.997	0.931	0.944	0.950

^a^n: noise level (range of 0, 1, 3 and 5%); rf: intensity uniformity (range of 0 and 20%)

**Table 2:** The results of GM, WM and CSF quantification in the high-resolution synthetic MRI (1x1x1mm^3^) at various parameter settings by using SPM8 software.

	**Accuracy**			**Sensitivity**			**Specificity**			**Similarity index**		
	**GM**	**WM**	**CSF**	**GM**	**WM**	**CSF**	**GM**	**WM**	**CSF**	**GM**	**WM**	**CSF**
**n0rf0**	0.954	0.978	0.970	0.786	0.800	0.600	0.970	0.998	0.977	0.746	0.881	0.433
**n1rf0**	0.975	0.987	0.983	0.878	0.869	0.934	0.985	1.000	0.984	0.866	0.929	0.672
**n3rf0**	0.988	0.994	0.991	0.941	0.940	0.964	0.993	0.999	0.992	0.937	0.963	0.847
**n5rf0**	0.987	0.992	0.992	0.922	0.948	0.953	0.994	0.996	0.993	0.930	0.954	0.871
**n1rf20**	0.975	0.987	0.983	0.881	0.869	0.937	0.985	1.000	0.984	0.868	0.930	0.678
**n3rf20**	0.985	0.993	0.989	0.920	0.937	0.943	0.992	0.999	0.990	0.923	0.962	0.803
**n5rf20**	0.987	0.992	0.991	0.924	0.950	0.944	0.994	0.996	0.993	0.932	0.955	0.858

n: noise level (range of 0, 1, 3 and 5%); rf: intensity uniformity (range of 0 and 20%)

### Clinical Image Data Analysis

**Fig 2:**
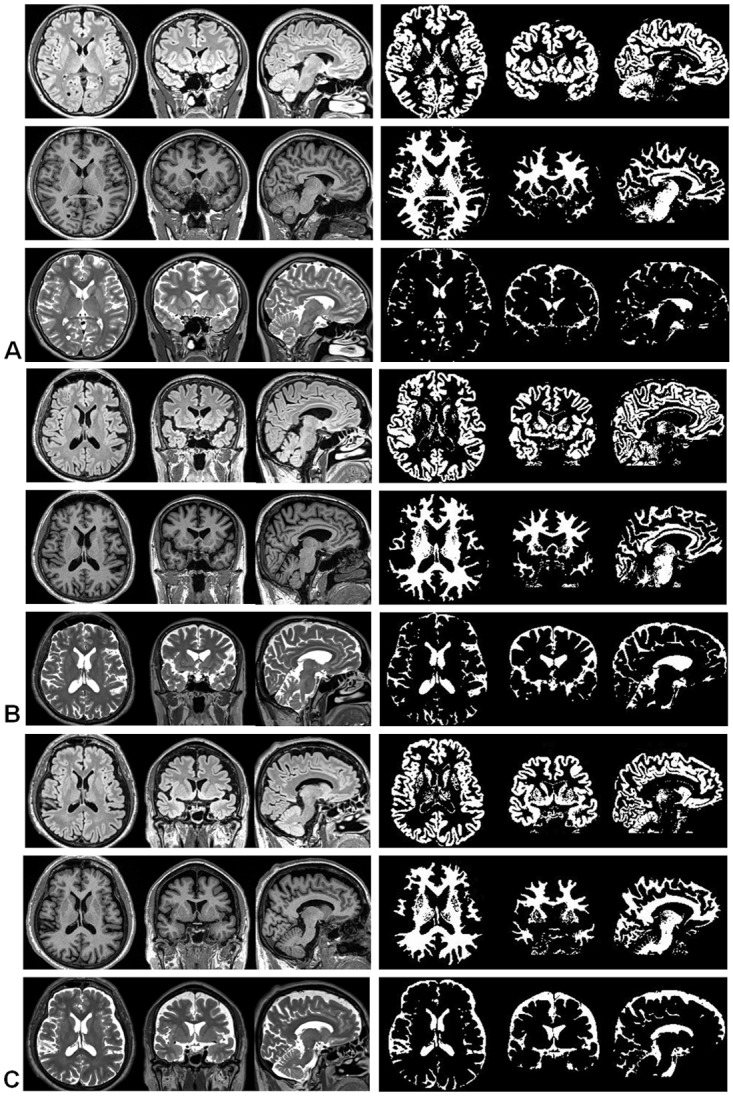
The results of brain classification images from 3D multispectral-multislice MRI. Left side reveals 3D multispectral MRI of FLAIR, T1WI and T2WI and right side is the classification images. Upper, middle and lower rows show GM, WM and CSF images. (A) A 20 year old young female with 587.2 ml, 433.6 ml and 154.8 ml of GM, WM and CSF, and 49.9%, 36.9% and 13.2% of GM, WM and CSF volume fractions. (B) A 60 year old healthy male with 636.0 ml, 587.3 ml and 326.8 ml of GM, WM and CSF, and 41.0%, 37.9% and 21.1% of GM, WM and CSF volume fractions. (C) A 76 year old dementia patient with 562.3 ml, 454.3 ml and 333.1 ml of GM, WM and CSF, and 41.7%, 33.7% and 24.7% of GM, WM and CSF volume fractions.

Effective classification and volume measurement was performed for three study groups consisting of 30 subjects. After the pre-processing step, the TRIO algorithm took approximately 30 seconds to complete classification processing of a multislice-multispectral 3DFT MRI data in MATLAB 7.12 (MathWorks, Inc. Natick, Massachusetts) running on an Intel 3.40 Giga-Hz CPU system with 8.00 Giga-Bytes of RAM memory. The time for generating the training data usually took less than another 30 seconds. Fig. 2 illustrates examples of GM, WM, and CSF segmented images from three study groups. Although no gold standard was available for in vivo studies, the quantitative data of the brain tissue and CSF volumes aligned with the brain morphometrics in these three study groups. As for the analysis of GM and WM volume measurements, the results revealed a larger reduction in the mean absolute volume of GM than that of WM in elderly volunteers compared to young adults, while an equal reduction in the mean absolute volume of GM and WM in dementia patients compared to healthy elderlies. As for analysis of brain volume fractions, the results showed a decrease in mean GM volume fraction and an increase in mean WM volume fraction in elderly volunteers compared to young adults. For dementia patients, reductions of the mean volume fractions were demonstrated in both GM and WM as compared to the elderly volunteers.

In an analysis of the reproducibility of the TRIO algorithm in brain volume quantification, the results showed very low intra- and inter-operator variability in measurements of the absolute volumes and volume fractions of cerebral GM, WM, and CSF in three study groups, as shown in Tables 3 and 4. Despite that the variability of CSF measurements was slightly higher than those of GM and WM in young adults, the mean coefficients of variation (CV) of the CSF volume and volume fraction were not larger than 0.45%. The mean CV values of the absolute volumes and volume fractions in cerebral GM, WM, and CSF were slightly higher in dementia patients than those of young adults and elderly volunteers, but which values were quite low in the range of 0.05% to 0.50%.

**Table 3:** GM, WM and CSF volume quantification in three groups of subjects by three measurements by (A) one operator and (B) three operators.

A	Measurements by one operator					
	Young adults		Healthy elderlies		Dementia	
	Mean	CV.%	Mean	CV.%	Mean	CV.%
GM	703.7±100.8	0.06±0.04	589.7±40.2	0.06±0.05	536.1±69.2	0.18±0.15
WM	522.7±87.3	0.03±0.02	511.4±25.1	0.05±0.06	477.8±64.8	0.19±0.21
CSF	157.8±33.1	0.30±0.23	209.5±35.7	0.07±0.06	260.2±62.3	0.20±0.18
GM+WM	1226.4±185.0	0.03±0.02	1101.1±48.4	0.01±0.01	1014.0±124.4	0.05±0.04

B	Measurements by three operators					
	Young adults		Healthy elderlies		Dementia	
	Mean	CV.%	Mean	CV.%	Mean	CV.%
GM	703.8±100.5	0.11±0.09	589.6±40.2	0.07±0.05	535.7±71.9	0.25±0.20
WM	522.8±87.3	0.06±0.06	511.6±25.0	0.06±0.05	478.3±64.9	0.23±0.25
CSF	157.7±33.3	0.45±0.34	209.3±35.8	0.20±0.28	260.1±62.4	0.24±0.22
GM+WM	1226.5±184.8	0.05±0.03	1101.2±48.2	0.03±0.05	1014.0±124.3	0.05±0.05

**Table 4:** Quantification of global GM, WM and CSF volume fractions in three groups of subjects by (A) one operator and (B) three operators.

A	Measurements by one operator					
	Young adults		Healthy elderlies		Dementia	
	Mean	CV.%	Mean	CV.%	Mean	CV.%
GM	50.9±1.4%	0.07±0.05	45.1±2.3	0.06±0.05	42.9±2.0%	0.26±0.19
WM	37.7±1.5%	0.03±0.02	39.0±1.9	0.06±0.06	36.7±3.3%	0.31±0.27
CSF	11.4±1.8%	0.30±0.23	15.9±2.3	0.07±0.06	20.4±3.5%	0.19±0.19
GM+WM	88.6±1.8%	0.04±0.03	84.1±2.3	0.01±0.01	79.6±3.5%	0.05±0.04

B	Measurements by three operators					
	Young adults		Healthy elderlies		Dementia	
	Mean	CV.%	Mean	CV.%	Mean	CV.%
GM	50.8±1.6	0.09±0.08	45.8±1.8	0.05±0.03	44.0±1.8	0.43±0.44
WM	37.9±2.0	0.05±0.03	38.8±1.7	0.08±0.11	36.2±3.2	0.50±0.68
CSF	11.3±1.8	0.43±0.30	15.4±2.0	0.17±0.25	19.7±3.5	0.31±0.37
GM+WM	88.7±1.8	0.05±0.04	84.6±2.0	0.03±0.04	80.3±3.5	0.07±0.08

## Discussion

The proposed TRIO algorithm re-invents wheel by looking into the feasibility of using multispectral remote sensing techniques to perform classification of multislice-multispectral brain MRI acquired with 3DFT high spatial-resolution imaging sequences. The preliminary results illustrated that the TRIO algorithm, made up of ICA, SVM, and IFLDA, could effectively enhance and improve weaknesses suffered from many currently being used segmentation techniques in clinical practice. First of all, the hybrid classifier provides a robust and consistent classification of brain MRI where the process of ICA is fully automated in enhancing the image contrasts of brain tissues with no operator intervention. The input to SVM only needs of support vectors selected by an operator for brain tissue classes of interest. The output of SVM serves as a large pool of training samples to be used as an initial set of training samples for iterative FLDA, which automatically classify whole high-resolution multislice-multispectral brain MRI data. The experimental results revealed extremely high reproducibility in classification of high-resolution synthetic MRI data, independent of the operator’s experience, by using the supervised trio-algorithm classifier.

Secondly, the TRIO algorithm, ICA, SVM, and FLDA provides the most benefit of operating segmentation in the native coordinate space. Such a hybrid classification approach can not only avoid the registration problems in the transformation to a standard coordinate space, but also preserves the high spatial-resolution image profiles without smoothing filtering during the coordinate transformation processing. This could explain the high accuracy in classification of multispectral synthetic MR data. In addition, the hybrid classifier provides a more effective tool in the segmentation of CSF, which had seldom been performed in most of the previous reports [3, 16, 17, 33]. There exists a wide range of various absolute volumes of GM, WM, and CSF between subjects, which may be dependent on age, gender, sex or other characteristics, such as the height and weight of the subjects [34–36]. An adequate measurement of CSF volume would be helpful in characterization of the brain structural alterations in specified diseases [37]. Our results showed the similarity indexes of CSF classification were in range of 0.949 to 0.965, which were as high as those of GM and WM in multispectral synthetic data, and also much higher than those by using SPM8. The results of the clinical image data analysis exhibited the evident differences of brain morphometry between normal young adults and other two challenging groups of healthy elderlies and dementia patients. As compared with the young adults, reduction of mean absolute GM/WM volumes and mean GM volume fraction, but no decline of mean WM volume fraction were illustrated in the healthy elderlies, which were compatible with the previous reports about the brain volume decline in aging [35,37]. There was an obvious decline of both mean absolute GM/WM volumes and volume fractions in the dementia patients. As compared with the healthy elderlies, the reduction of absolute and fractional volumes in the dementia patients was aligned with those reported in the literatures [35, 37, 38]. Although no gold standard available in the in vivo study for comparison, the quantitative assessments of brain structural alternations in the challenging groups would effectively demonstrate the performance of the TRIO algorithm in clinical practice.

Thirdly, there is no need for probability maps to initiate segmentation. The atlas-based approaches, such as SPM tool packages, are initially created by a default standard tissue probability atlas, and are helpful in yielding accurate and consistent segmentation of multispectral brain MRI. But, there are drawbacks including the prior probability images tending to be very blurred and uninformative, as well as registration and labeling errors. Most of the default probability maps are only feasible in segmentation of normal brain tissue [9]. The approaches have commonly been applied to segmentation of GM, WM, and CSF in healthy subjects, but are not for segmentation of the diseased brain. Instead of using probability maps to initialize classification, the hybrid classifier works with a small set of the manually labeled training samples based on the prior knowledge of operators. The experimental results illustrated that process achieves effective classification of GM, WM, and CSF not only for normal young adults, but also for healthy elderlies and dementia patients.

Finally, the process of the TRIO algorithm is simple and the operator burden is minimal. Usually, high-level segmentation techniques need a longer processing time and typically not capable of performing whole multislice-multispectral MRI data in a one shot operation. FAST uses a MRF model and Expectation-Maximization (EM) algorithm to account for the pixel intensity information with the neighborhood spatial information for the outperforming segmentation [14]. However, in the segmentation of multispectral images, the information from each channel of multispectral MRI is taken from a slice of brain at a time and the segmentation of a single image is performed using only three channels of brain images [16]. Additionally, the computational complexity may also impair its performance [16, 17]. In the TRIO algorithm, the training samples of 3x3 pixels of GM, WM, CSF, and BG were manually depicted from one slice of multislice-multispectral MRI for SVM to produce a large pool training samples from this particular image slice, which serve as the training samples for IFLDA. The whole process takes about 30 seconds to complete classification. The time consumption is considerably less than other segmentation tools, such as SPM8 and FAST, which took minutes and hours to complete a segmentation process [17].

Though the proposed method could perform a robust classification of multislice-multispectral MRI of normal brain with operator independent results, fully automated techniques would be superlative for image segmentation and classification in order to remove any operator intervention as well as to efficiently enhance the image processes. The TRIO algorithm would be potentially extendable to an unsupervised method for labeling the training sample automatically. Nevertheless, the issues will not be discussed in this paper. There is the lack of a gold standard for in vivo MRI data available for testing the performance of a given method. Recently, some standard test image data sets, such as IBSR, have been proposed for the evaluation of sensitivity of the single-image segmentation techniques. However, there is still a lack of the standard test multi-spectral image data available for assessment the accuracy of the multispectral classifiers. We had attempted to create a gold standard for clinical data by performing manual segmentation of GM, WM, and CSF in multispectral brain MRI. However, the results showed lower similarities of intra- and inter-operator measurements in manual segmentation of multispectral brain MRI (the results were not reported in this paper). We believed that manual segmentation of multispectral brain MRI may not show sufficient amount of reliability. Therefore, we emphasized the issues of consistency in brain segmentation rather than accuracy. In our experiments, the aim was focused on testing the reproducibility, instead of accuracy, of the TRIO algorithm for multislice-multispectral MRI.

## Conclusions

This study presents a new application of a supervised hybrid classifier for the classification of multislice-multispectral MRI and also demonstrates its clinical feasibility in volume assessment of brain tissue in different subject groups. The proposed method intelligently integrates three different algorithms, ICA, SVM, and IFLDA. Based on the multispectral remote sensing techniques, this TRIO algorithm can perform robust classification of brain MR images with significantly reduced computational complexity while avoiding the registration problems in transformation to a standard coordinate space. In addition, there is also no need for probability maps in segmentation. The method has shown to be promising and practicable by experiments in cross-sectional and longitudinal studies of brain volumetric analysis in different subject groups, particularly some challenging subjects. Since the experimental results demonstrated the clinical feasibility in classification of GM, WM, and CSF by manually labeling the target normal brain tissues, the TRIO algorithm derived from multispectral remote sensing techniques might also be potentially expandable and applicable to automated classification of multislice-multispectral brain MRI as well as simultaneous segmentation of brain normal tissues and pathologies to further enhance clinical practicability.
